# SSAlign: fast and sensitive protein structure search at scale

**DOI:** 10.1093/bioinformatics/btag789

**Published:** 2026-10-26

**Authors:** Lei Wang, Xuchao Zhang, Yan Wang, Zhidong Xue

**Affiliations:** School of Software Engineering, Huazhong University of Science and Technology, Wuhan 430074, China; School of Software Engineering, Huazhong University of Science and Technology, Wuhan 430074, China; School of Software Engineering, Huazhong University of Science and Technology, Wuhan 430074, China; College of Life Science and Technology, Huazhong University of Science and Technology, Wuhan 430074, China; School of Software Engineering, Huazhong University of Science and Technology, Wuhan 430074, China

## Abstract

**Motivation:**

Predicted protein structure resources now contain hundreds of millions of models, turning structural comparison from a pairwise alignment task into a database-scale retrieval problem. Exhaustive structural aligners are computationally impractical at this scale, whereas fast structural-alphabet searches may discard remote or low-complexity homologs during prefiltering. Database-scale search therefore requires methods that combine rapid retrieval with sensitivity to global structural similarity.

**Results:**

We present SSAlign, a protein structure search method that integrates SaProt sequence-structure embeddings, entropy-reduced dense retrieval, an SS-score predictor, and two-stage alignment refinement. In benchmarks on the Swiss-Prot, SCOPe40, and AFDB50 datasets, SSAlign showed competitive alignment quality and returned more TM-align-supported hits than Foldseek in recall-oriented evaluations. On AFDB50, 1000 queries against 53.7 million structures completed in 3150 s on CPUs and 2290 s with GPU prefiltering, corresponding to 103-fold and 142-fold speedups over Foldseek, respectively. On SCOPe40, SSAlign increased the area under the curve (AUC) by 20.2% at family level and 33.3% at superfamily level. In simple-fold antimicrobial peptide case studies, SSAlign retrieved candidates that were not returned by Foldseek under the tested settings, indicating its utility as a fast candidate-generation tool for proteome-scale structural search.

**Availability and implementation:**

The source code for SSAlign is publicly available at https://github.com/ISYSLAB-HUST/SSAlign. The software version and test data used in this study are archived on Zenodo at https://doi.org/10.5281/zenodo.23432919. Datasets and prebuilt indices can be downloaded from http://bioinfo.isyslab.info/ssalign/download/section/ssalign/.

## 1 Introduction

Identifying homologous proteins is important for functional annotation, evolutionary analysis, and structure-guided biological discovery (Penny *et al.* 1982, Helbert *et al.* 2019, Torres *et al.* 2021). While sequence-based methods remain widely used, they often miss distant homologs. Protein structures are generally more conserved than amino acid sequences, so structure comparison can reveal relationships that sequence comparison misses. Recent progress in structure prediction has greatly expanded the amount of available structural data. The AlphaFold Protein Structure Database now provides more than 214 million predicted structures (Varadi *et al.* 2024), and ESMFold has added more than 617 million metagenomic protein models through the ESM Atlas (Lin *et al.* 2023). With the advent of AlphaFold3 (Abramson *et al.* 2024), both the size and complexity of these resources continue to increase. As a result, comparing hundreds of millions of protein structures has become a major computational bottleneck.

Sequence-based approaches such as MMseqs2 (Steinegger and Söding 2017), BLASTp (Altschul *et al.* 1990), and DIAMOND (Buchfink *et al.* 2015) have long been standard tools for large-scale homology detection because of their speed and scalability. However, sequence similarity alone often fails to uncover remote evolutionary relationships, since protein tertiary structures are generally more conserved than primary amino acid sequences (Hasegawa and Holm 2009). Structure alignment tools such as TM-align (Zhang and Skolnick 2005) are more sensitive for distant homolog detection, but their computational cost limits their use at database scale. For example, searching a database of 100 million protein structures with a single query using TM-align can take more than a month (van Kempen *et al.* 2024). To improve efficiency, Foldseek encodes protein backbone geometry into a 20-letter structural alphabet and performs rapid k-mer-based search and alignment (van Kempen *et al.* 2024). However, the continued growth of predicted protein structure databases may still make large-scale structural search expensive. In addition, discretized structural representations can lose spatial detail during prefiltering, which may cause some structurally similar targets to be discarded before alignment.

Protein language models (PLMs) provide another route for protein representation learning. By training transformer models on large protein sequence corpora, PLMs generate embeddings that capture evolutionary and structural information and can support remote homolog retrieval without explicit alignment. This idea has already been explored in sequence retrieval. Dense homolog retriever (DHR) (Hong *et al.* 2025), e.g. uses PLM embeddings for dense similarity search and improves remote homolog detection over traditional methods. However, DHR relies on large-scale supervised contrastive learning. This prior work motivates retrieval strategies that use PLM representations without requiring large supervised training sets. PLMs have also become more diverse in their use of structural information. Earlier models such as UniRep (Alley *et al.* 2019) and ProtTrans (Elnaggar *et al.* 2022) were widely used for sequence modeling, and the ESM family (Rives *et al.* 2021, Lin *et al.* 2023, Hayes *et al.* 2025) further improved representation quality through larger-scale training. More recent models incorporate explicit structure: SaProt (Su *et al.* 2024) generates structure-aware (SA) embeddings by discretizing 3D features, whereas ESM-3 (Hayes *et al.* 2025) jointly models sequence, structure, and function. Given its balance between structural representation and computational cost, SaProt was selected as the main encoder in SSAlign.

We developed SSAlign, a structural retrieval system that combines SaProt embeddings with dense vector search. Here, dense vector search means representing each protein as a fixed-length, real-valued embedding and retrieving candidate proteins by comparing query and database embeddings using a vector similarity measure. SSAlign uses an L2-normalized FAISS IndexFlatIP index for exact inner-product search, which is equivalent to cosine-similarity search after normalization. The retrieved candidates are then refined with a two-stage scoring procedure. As shown in Fig. 1, the pipeline first encodes each protein with Foldseek’s structural encoder, which produces 3Di tokens, and with SaProt, which produces sequence embeddings. The entropy reduction module (ERM) then combines these signals into a unified embedding for both queries and database entries through spatial redistribution and dimensionality reduction. Candidate homologs are retrieved by vector similarity search and scored by the SS-score predictor. For candidates below a preset confidence threshold, the SAligner module performs global alignment using an accelerated Needleman–Wunsch algorithm with a 3Di substitution matrix. SSAlign runs on either multi-GPU or CPU-only hardware and can search tens of millions of protein structures within batch runtimes suitable for database-scale screening. Relative to Foldseek, it provides a speedup of approximately two orders of magnitude in our AFDB50 benchmark while improving several recall-oriented metrics. On SCOPe40, SSAlign increases family-level AUC by 20.2% and superfamily-level AUC by 33.3%. On Swiss-Prot, it returns more TM-align-supported matches, with cumulative TM-score and RMSD trends close to TM-align-ranked references. SSAlign also improves retrieval for the tested small peptides and other simple-fold proteins, a class of cases in which Foldseek returned fewer matches under our settings.

**Figure 1:**
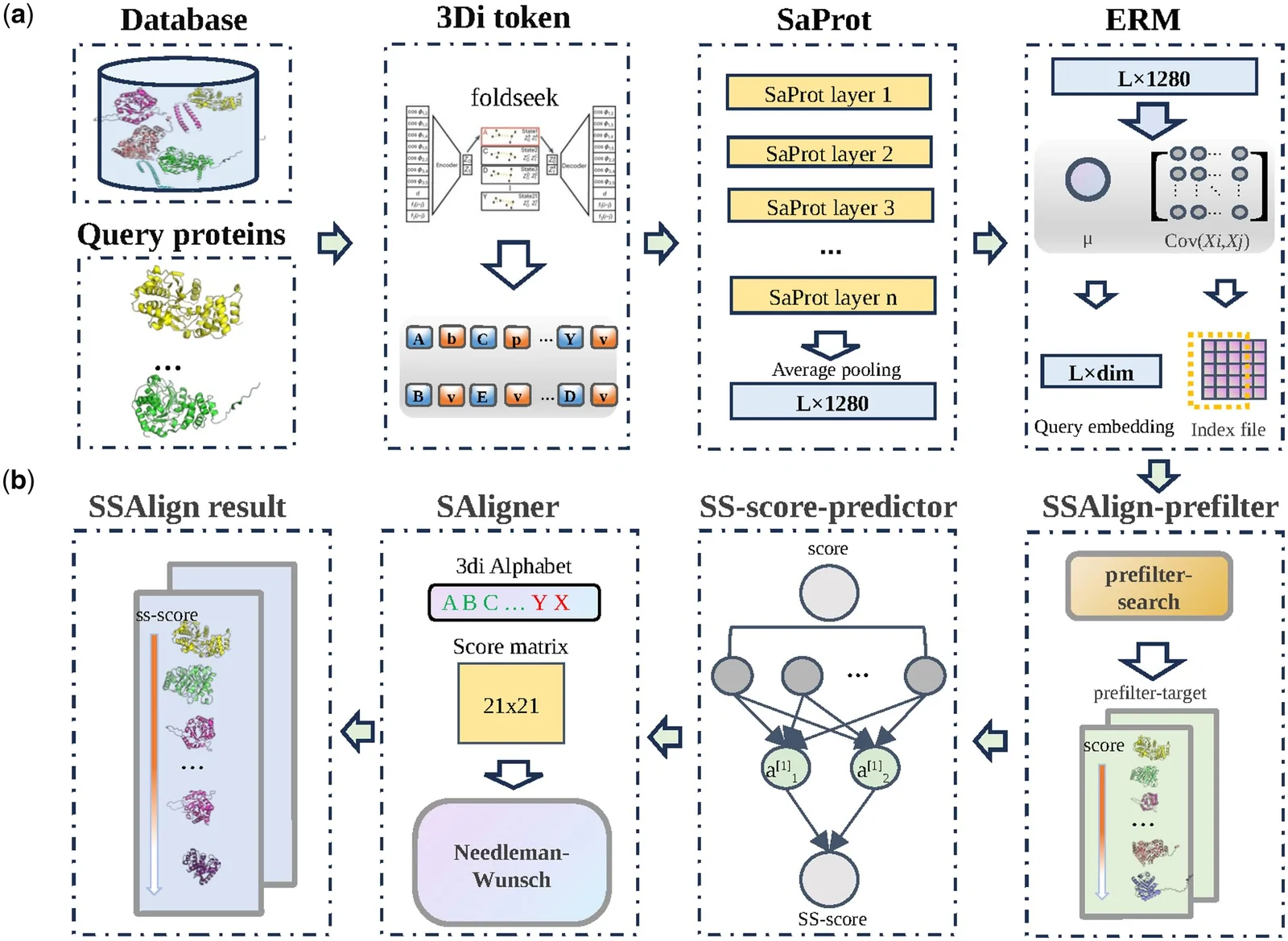
Overview of the SSAlign workflow. (a) Query and database structures are converted to 3Di tokens, encoded by SaProt, and transformed by ERM to generate query embeddings and the searchable vector index. (b) SSAlign-prefilter retrieves candidate proteins from the index. The SS-score predictor converts the prefilter similarity into an estimate of global structural similarity; candidates below the preset confidence threshold are further rescored by SAligner using an accelerated Needleman–Wunsch algorithm and a 21-state 3Di substitution matrix.

## 2 Methods

### 2.1 Structure-aware protein language model

SaProt is a large-scale PLM trained on protein sequence and structural data. To incorporate structural information, we used the 3Di structural discretization module from Foldseek to convert 3D protein structures into discrete structural tokens, which were then combined with residue-level tokens to construct a SA vocabulary. This vocabulary serves as input to a conventional PLM architecture for unsupervised training on large-scale SA-token sequences.

SaProt has 650 million parameters and was trained on a large protein structure dataset. Training was conducted over 3 months using 64 NVIDIA A100 80 GB GPUs, with total computational cost comparable to that of ESM-1b (Rives *et al.* 2021, Su *et al.* 2024). Built upon the ESM architecture, SaProt incorporates structural information through pretraining on SA tokens and has been evaluated across multiple downstream tasks. To determine the representation used in SSAlign, we compared embeddings from the final (33rd) layer with embeddings obtained by averaging the first and final layers. The final-layer embeddings gave higher overlap rates in our validation experiments (Table 3, available as supplementary data at *Bioinformatics* online) and were therefore used in all subsequent experiments.

### 2.2 Entropy reduction module

To adapt SaProt embeddings for similarity search in the SSAlign-prefilter stage, one option is to convert the model into a dual encoder trained with contrastive learning to encode queries and targets separately (Hong *et al.* 2025). However, this strategy is computationally expensive. Another option is to use flow-based models to calibrate vector distributions, as has been done for semantic retrieval with BERT embeddings. Flow-based models are also costly because their invertible layers limit the transformation capacity of each layer and require deep architectures for adequate fitting. Instead, we developed the lightweight ERM. SaProt embeddings exhibit inter-dimensional correlation and unequal variance, so some dimensions can dominate cosine-similarity calculations (Eq. (1)) and distort retrieval results. Together, these effects produce an anisotropic embedding distribution in which some directions are overrepresented. ERM is designed to reduce this bias and make similarity comparisons more reliable


(1) $$\text{cos}(x,y)=\frac{\sum_{i=1}^{d}x_{i}y_{i}}{\sqrt{\sum_{i=1}^{d}x_{i}^{2}}\sqrt{\sum_{i=1}^{d}y_{i}^{2}}}$$

ERM uses a linear transformation to reduce inter-dimensional correlations in the data (Algorithm 1). This makes cosine-similarity calculations less dominated by redundant or high-variance dimensions, while standardization balances the contribution of each dimension to similarity measurements. ERM approximately whitens the embedding distribution and reduces anisotropy. The transformation is intended to reduce bias in distance (or angular) calculations caused by anisotropic data distributions. In our retrieval experiments, this produced more discriminative vector distances.

Algorithm 1. ERM workflow

**Require:** Existing embeddings ${\left\{x_{i}\right\}}_{i=1}^{N}$ and reserved dimensionality *k*

1. Compute μ and Σ of ${\left\{x_{i}\right\}}_{i=1}^{N}$
2. Compute $U,\Lambda ,U^{T}=\text{SVD}(\Sigma )$
3. Compute $W=(U\sqrt{\Lambda^{-1}})[\cdot ;k]$
4. **for**  i=1,2,…,N  **do** 
5.    ${\tilde{x}}_{i}=(x_{i}-\mu )W$
6. **end for** 
7. Transformed embeddings ${\left\{{\tilde{x}}_{i}\right\}}_{i=1}^{N}$

For AFDB50, the mean and covariance are accumulated incrementally rather than by loading all embeddings at once. If *n* samples have running mean $\mu_{n}$ and population covariance $\Sigma_{n}$, a new vector $x_{n+1}$ is incorporated as


(2) $$\mu_{n+1}=\frac{n}{n+1}\mu_{n}+\frac{1}{n+1}x_{n+1},$$

(3) $$\Sigma_{n+1}=\frac{n}{n+1}\left(\Sigma_{n}+\mu_{n}\mu_{n}^{T}\right)+\frac{1}{n+1}x_{n+1}x_{n+1}^{T}-\mu_{n+1}\mu_{n+1}^{T}.$$

This calculation stores the d×d covariance matrix and *d*-dimensional mean, giving $O(d^{2})$ working memory independent of the number of database entries.

By retaining only the leading dimensions of the transformed embeddings, ERM also enables dimensionality reduction. Reducing the embeddings to 512 dimensions preserved screening performance in the tested settings and slightly improved overlap at some top-*k* thresholds (Table 1). At 1280 dimensions, ERM increased the overlap rate by approximately 32 percentage points, while the 512-dimensional embeddings achieved comparable performance. After applying ERM, reducing the embedding dimensionality from 1280 to 512 did not materially reduce retrieval overlap. For example, the overlap rates remained similar at top-*k *= 1000 and 2000 (85.36/89.94 for 1280 dimensions versus 83.70/89.36 for 512 dimensions), and at top-*k *= 4000 the 512-dimensional embeddings showed slightly higher overlap (92.86 versus 92.41). The reduced-dimensional embeddings also lowered memory usage and runtime, with approximately 50% less memory consumption. We therefore use ERM-processed 512-dimensional embeddings as the protein representation in SSAlign.

**Table 1:** Overlap between Foldseek and SSAlign-prefilter results with and without ERM.

Type	Dimension	Top-*k*	Overlap rate (%)
**Without ERM**	1280	1000	53.32
		2000	57.69
		4000	62.17
**With ERM**	1280	1000	85.36
		2000	89.94
		4000	92.41
**With ERM**	512	1000	83.70
		2000	89.36
		4000	92.86

Consequently, SSAlign-prefilter selects the 512-dimensional embeddings processed by ERM as the feature representation for proteins.

### 2.3 SSAlign database

To reduce the memory cost of storing protein embeddings, we built the SSAlign index with the FAISS library (Douze *et al.* 2026). FAISS is designed for large-scale vector retrieval on both CPUs and GPUs. On AFDB50, which contains about 50 million sequences, FAISS provided the vector-search backend used in the runtime benchmark. To support rapid retrieval of target protein 3Di sequences for SAligner, we also built an indexed 3Di sequence database for all proteins in AFDB50. We constructed complete SSAlign databases for Swiss-Prot, SCOPe40, and AFDB50 with two components:

1. Vector index database (for SSAlign-prefilter): built using the FAISS library.
2. 3Di sequence database (for SAligner retrieval): enables target protein 3Di sequence lookup with reduced memory use.

Both components were designed to limit memory consumption during database search.

### 2.4 SS-score-predictor

The SSAlign-prefilter stage ranks candidates by vector similarity, typically cosine similarity, but this score is not itself a structural similarity metric. TM-score (Zhang and Skolnick 2004) is widely used to evaluate protein structural similarity. It is calculated from *C*${\;}_{\alpha }$ distance deviations while accounting for protein size and shape, making it less sensitive to protein length than RMSD alone. However, TM-score computation requires comparing entire structures, which is costly for large-scale alignment. The avg_TM-score is defined as the average of the normalized TM-scores for both query and target chains:


(4) $$\text{avg}\_\text{TM-score}=\frac{1}{2}\left({\text{TM-score}}_{\text{query}}{+\text{TM-score}}_{\text{target}}\right)$$

To rapidly assess structural similarity between proteins, SSAlign uses an evaluation metric called the SS-score. The SS-score predictor maps the cosine similarity obtained at the prefilter stage to an estimate of avg_TM-score using a dataset-specific linear relation. Coefficients were determined separately for Swiss-Prot and SCOPe40 and are reported in Table 4, available as supplementary data at *Bioinformatics* online. For the Swiss-Prot dataset with 512-dimensional embeddings, the relation is:


(5) SS-score=0.69*Cosine_Similarity+0.39

The Pearson correlation coefficient between the SS-score and avg_TM-score was 0.69 for the evaluated Swiss-Prot protein pairs. Unlike Foldseek, which focuses on local similarity, the SS-score is designed to approximate global structural similarity in a way that is closer to TM-score.

### 2.5 3Di sequence-based SAligner

Needleman–Wunsch is a widely used global alignment algorithm for sequence comparison (Needleman and Wunsch 1970). As a dynamic-programming method, it identifies an optimal global alignment between two sequences by maximizing similarity or minimizing differences. Compared with local alignment algorithms such as Smith–Waterman (Smith and Waterman 1981, Pearson 1991), global alignment is more appropriate for full-length evolutionary and structural comparisons.

For protein sequence alignment, substitution matrices such as BLOSUM62 (Henikoff and Henikoff 1992, Eddy 2004, Styczynski *et al.* 2008) or PAM250 (Dayhoff *et al.* 1978, Singh *et al.* 2014) quantify the likelihood of amino acid replacements. Because Foldseek discretizes protein structures into 3Di sequences, we implemented a Needleman–Wunsch variant for 3Di sequence alignment using a 21-state structural substitution matrix that includes the 20 3Di states and an unknown state. SAligner uses affine gap penalties with a gap-opening score of −10 and a gap-extension score of −1. Its score-only dynamic-programming kernel is compiled to native code with Numba using fastmath, disabled bounds checking, and function caching, and it uses linear memory with respect to the length of the second sequence. The codebase also provides a numba.pycc ahead-of-time compilation wrapper to avoid the startup overhead of just-in-time (JIT) compilation. Batch rescoring is parallelized across candidate alignments (default: 64 concurrent tasks), and the resulting SAligner score is used as a second-stage rescoring signal.

### 2.6 Hyperparameter configuration

The two-stage design of SSAlign allows sensitivity and runtime to be tuned through the number of retained prefilter candidates, the rescoring threshold, and the execution backend. The reported experiments used --prefilter_target 2000, --prefilter_threshold 0.3, and --max_target 1000; detailed command-line parameters are provided in Table 5, available as supplementary data at *Bioinformatics* online. Figure 6, available as supplementary data at *Bioinformatics* online, shows how the *prefilter_threshold* parameter affects the precision–recall tradeoff. Lower thresholds increase sensitivity but require more correction through SAligner realignment and rescoring.

### 2.7 Datasets and benchmark

We assessed structural alignment accuracy from the spatial consistency of aligned protein pairs. This evaluation does not rely on external classification systems that may themselves depend on specific sequence or structural alignment tools (Zhang and Skolnick 2005). TM-score serves as a global measure of alignment quality and is sensitive to coverage, ranging from 0 to 1, where 1 indicates a perfect structural match. The TM-score range of [0.5, 1] is especially relevant because it reflects the sensitivity of detecting structurally similar proteins (Xu and Zhang 2010). Since TM-score can be normalized by the length of either protein in the pair, we used the average of the two normalizations (avg_TM-score) to reduce bias from large length differences. Root mean square deviation (RMSD), normalized by the number of aligned residue pairs, provides a complementary measure of local spatial deviation between corresponding atomic positions.

For our Swiss-Prot benchmark, we used Swiss-Prot entries (The UniProt Consortium 2021) and their corresponding structures from the AlphaFold Protein Structure Database (Varadi *et al.* 2024). We randomly selected 100 query proteins and evaluated tool performance using both TM-score and RMSD metrics calculated by TM-align. The evaluation procedure consisted of:

1. Executing structural alignment tools.
2. Calculating TM-scores for all generated alignments using TM-align.
3. Ranking results by each tool’s native metrics (e.g. E-value, SS-score).
4. Additionally ranking alignments by TM-align calculated avg_TM-score.
5. Finally, computing cumulative avg_TM-score and RMSD values for both ranking methods.

This setup evaluates both alignment accuracy and retrieval efficiency. The SCOPe dataset (Chandonia *et al.* 2017, 2019) provides a curated hierarchical classification of protein structural domains based on structural and evolutionary relationships. We used the SCOPe40 dataset, which contains 11 211 non-redundant protein sequences clustered at 40% sequence identity from SCOPe 2.01. Based on the known hierarchical relationships, we defined matches within the same family or superfamily (Chandonia *et al.* 2022), or those with avg_TM-score ≥0.5 up to the first false positive (FP), as true positives (TPs), while matches not sharing the same fold with avg_TM-score <0.5 were classified as FPs. Benchmarking was performed by ranking results according to each tool’s native metrics and measuring, for each query, the fraction of TPs retrieved before encountering the first FP. We conducted an all-versus-all search after excluding the 13 simple-fold cases listed in Table 1, available as supplementary data at *Bioinformatics* online, from this benchmark.

For large-scale performance testing, we used the AFDB50 database containing 53 665 860 protein structures. We built complete FAISS index files for this database and used AFDB50 for the runtime benchmark. We also applied the Swiss-Prot evaluation procedure to AFDB50.

## 3 Results

We benchmarked SSAlign for runtime, alignment quality, and retrieval of homologs with repetitive or simple folds. Specifically, we evaluated large-scale search efficiency, agreement with TM-align-based structural similarity measures, and performance on cases for which Foldseek returned few or no homologous matches under the tested settings.

### 3.1 Order-of-magnitude acceleration in structure search

We evaluated search speed on the AFDB50 dataset by randomly selecting 1000 proteins as queries and comparing SSAlign with Foldseek under controlled conditions. The benchmark system was equipped with three NVIDIA RTX A6000 GPUs, each with 48 GB of memory, two Intel Xeon Gold 6133 processors operating at 2.50 GHz, and 256 GB of system RAM. SSAlign was run with --prefilter_target 2000, --prefilter_threshold 0.3, --max_target 1000, --nproc 64, and --mode 1, using either CPU or GPU prefiltering. Foldseek was executed with default parameters plus --threads 64. TM-align was excluded from timing comparisons because its estimated execution time at this scale exceeds 1 month.

SSAlign was approximately two orders of magnitude faster than Foldseek on AFDB50 under the tested settings (Table 2). Foldseek required 325 081 s (approximately 90 h) to complete the 1000-query benchmark, whereas SSAlign completed the same workload in 3150 s on CPU and 2290 s with GPU acceleration. This corresponds to a 103× speedup on CPU and a 142× speedup on GPU. The CPU result indicates that most of the acceleration comes from the retrieval strategy and pipeline design rather than from GPU acceleration alone.

**Table 2:** Runtime comparison and speedup of SSAlign versus Foldseek on the AFDB50 dataset (1000 queries).

	**Time (s)**		**Speedup (** × **)**	
Method/stage	CPU	GPU	CPU	GPU
**Foldseek (easy-search)**	325 081	–	1.0	–
**SSAlign**				
** Preload**	634	898		
** Prefilter**	1645	521		
** SAligner**	871	871^a^		
** Total**	3150	2290	103.2	141.9

a The SAligner stage is executed on the CPU in both configurations.

The stage-level timings in Table 2 indicate the main contributors to this runtime reduction. GPU acceleration mainly shortened the Prefilter stage, reducing its runtime from 1645 s to 521 s (∼3.2× faster). Part of this benefit was offset by a longer Preload time (898 s versus 634 s), because the large index files must be transferred to GPU memory. The SAligner step, which runs on CPU in both settings, took 871 s, indicating that the prefilter limited the number of candidates passed to alignment. Thus, the main retrieval workflow can run on CPU-only hardware, with additional acceleration available from GPUs.

### 3.2 Comparative benchmarking of SSAlign against Foldseek and TM-align

We systematically benchmarked SSAlign, Foldseek, and TM-align on three widely used structural alignment datasets: Swiss-Prot, SCOPe40, and AFDB50. The term SSAlign-prefilter refers to the first-stage output of SSAlign prior to final scoring. Detailed evaluation procedures are described in Section 2.7.

#### 3.2.1 Benchmarking on the Swiss-Prot dataset

On the Swiss-Prot dataset, we randomly selected 100 query proteins and performed a reference-free evaluation using the cumulative TM-score and RMSD of retrieved protein pairs. Detailed cumulative-score and differential-hit analyses are provided in Figs 1 and 2, available as supplementary data at *Bioinformatics* online. When results were ordered by TM-align-derived avg_TM-score, SSAlign (including SSAlign-prefilter) remained close to the TM-align-ranked reference in cumulative avg_TM-score and RMSD, while the Foldseek curve decreased more rapidly, indicating that Foldseek returned fewer structurally similar targets in this benchmark. When results were ordered by each tool’s native score (E-value or SS-score), the TM-align-derived ranking remained the reference with the highest cumulative scores, and Foldseek showed slightly higher cumulative scores than SSAlign. However, SSAlign returned more matched protein pairs than Foldseek, and these additional pairs retained high cumulative TM-scores and low RMSDs, consistent with higher recall with limited loss of alignment quality.

**Figure 2:**
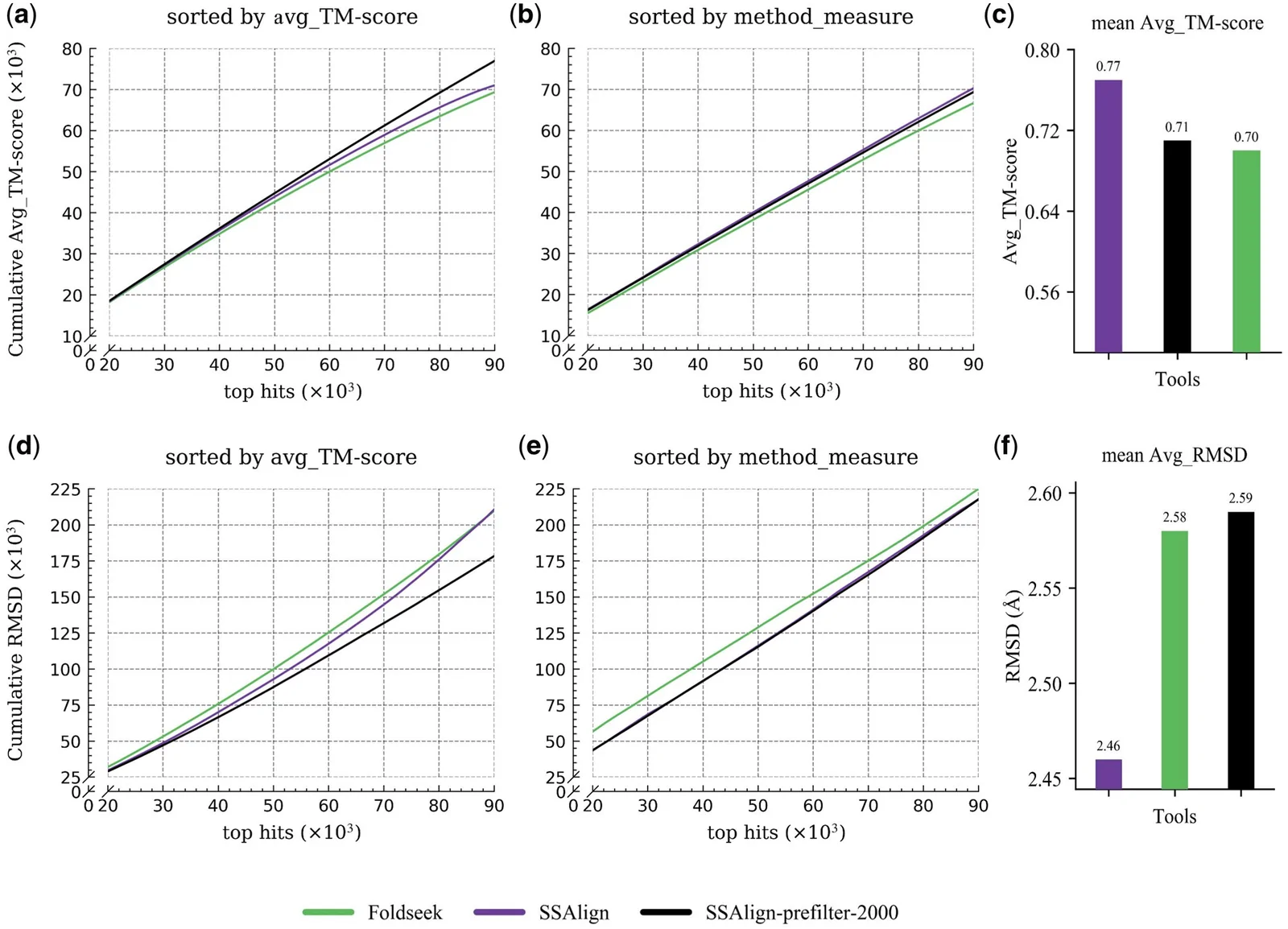
Comparative performance of structural alignment methods on the AFDB50 dataset. The benchmark used 100 query proteins and a database of 53 665 860 entries. (a, d) Cumulative avg_TM-score and RMSD for three methods when results are ordered by TM-align-derived avg_TM-score. (b, e) The same evaluation when results are ordered by each tool’s native score (E-value or SS-score). (c, f) Average scores of the hits returned by each tool across the 100 queries. Truncated curves indicate that a method returned fewer hits.

#### 3.2.2 Benchmarking on the SCOPe40 dataset

We next assessed homology detection on the SCOPe40 dataset, which provides hierarchical family and superfamily annotations based on structural similarity (Fig. 3, available as supplementary data at *Bioinformatics* online). SSAlign and its prefilter module retrieved more TP matches than Foldseek at both classification levels. The prefilter stage alone also separated positives from negatives, indicating that the embedding space captures discriminative structural information. Although SSAlign returned fewer total hits than TM-align, because TM-align performs exhaustive scoring and often yields redundant high-scoring matches, SSAlign achieved comparable precision–recall trends and higher recall than Foldseek. Quantitatively, SSAlign increased the area under the curve (AUC) at the family level from 0.213 (Foldseek) to 0.256 (+20.19%) and at the superfamily level from 0.186 to 0.248 (+33.33%).

**Figure 3:**
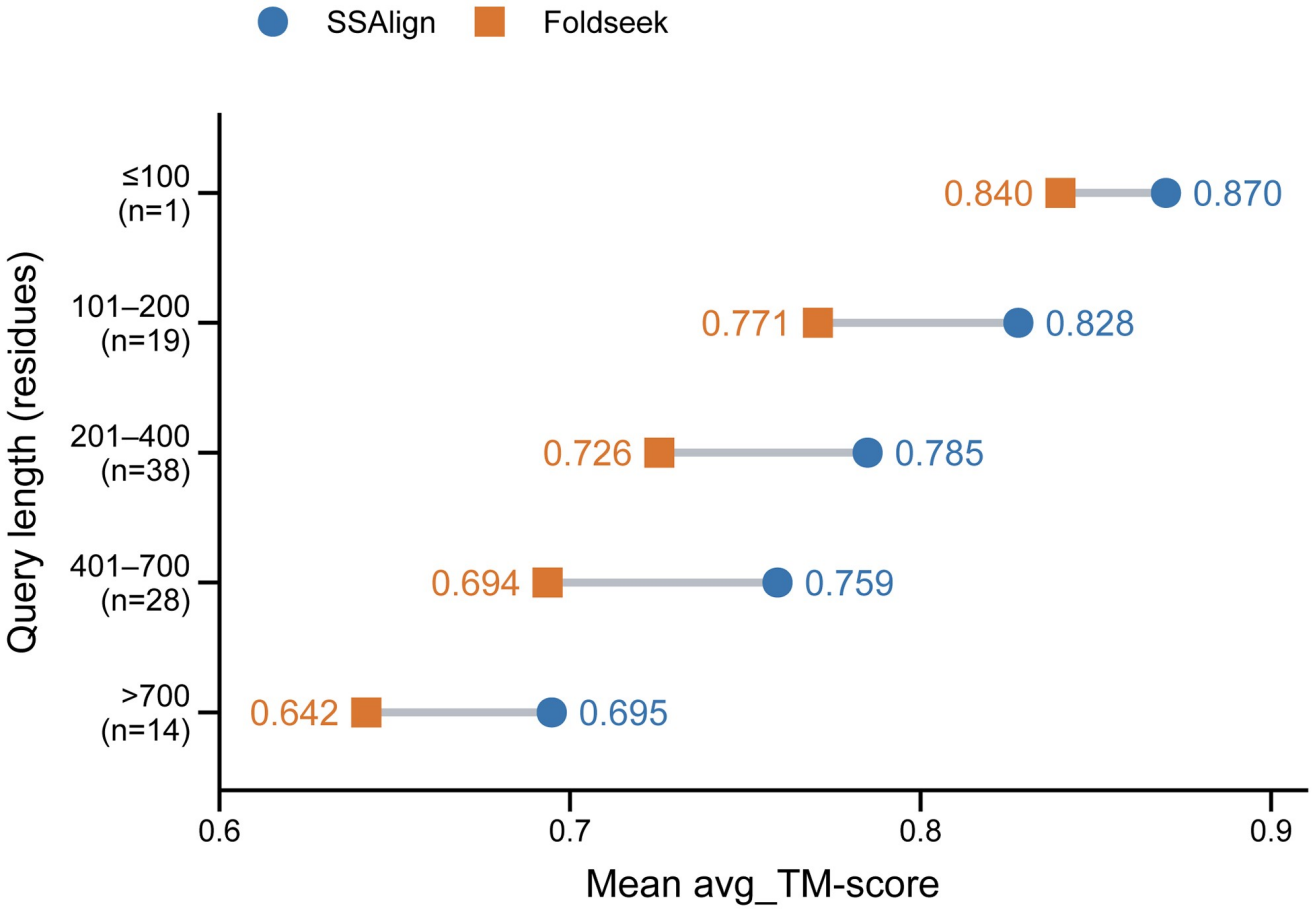
Comparison of SSAlign and Foldseek across protein lengths in the AFDB50 benchmark. Points indicate the mean avg_TM-score obtained by each method within each query-length group; gray lines connect results for the same group, and *n* denotes the number of queries. The analysis comprises the 100 queries selected for detailed structural evaluation. Across these queries, the mean avg_TM-score was 0.774 for SSAlign and 0.715 for Foldseek.

#### 3.2.3 Benchmarking on the AFDB50 dataset

For the AFDB50 benchmark, we first measured runtime with 1000 query tasks using SSAlign and Foldseek. Because TM-score and RMSD are expensive to compute at this scale, we then selected the 100 queries for which Foldseek returned the largest number of hits and evaluated the retrieved pairs with cumulative avg_TM-score and RMSD. As shown in Fig. 2a and d, when results were ordered by TM-align-derived avg_TM-score and compared at matched top-hit counts, SSAlign (including SSAlign-prefilter-2000) showed higher cumulative avg_TM-score and lower RMSD than Foldseek. When results were ordered by each tool’s native score (E-value or SS-score; Fig. 2b and e), SSAlign achieved higher cumulative avg_TM-score and lower RMSD than Foldseek, consistent with the re-ranking effect of SAligner. Figure 2c and f further shows that the hits returned by SSAlign had, on average, an approximately 10% higher avg_TM-score and an approximately 4.6% lower RMSD. The method-specific comparison in Fig. 4, available as supplementary data at *Bioinformatics* online, shows that although the Foldseek-only set (Foldseek SSAlign) is larger than the SSAlign-only set (SSAlign Foldseek), the SSAlign-only matches had higher TM-align-derived structural similarity under the same top-hit condition. Among hits retrieved uniquely by each method, the SSAlign-only hits showed an approximately 16.9% increase in avg_TM-score and an approximately 6.3% decrease in RMSD relative to the Foldseek-only hits.

To examine whether the result depended on protein size, we stratified the 100 AFDB50 evaluation queries by query length (Fig. 3). SSAlign had a higher mean avg_TM-score than Foldseek in every reported bin, with differences ranging from 0.030 for proteins of at most 100 residues to 0.066 for proteins of 401–700 residues. Across all 100 queries, the mean avg_TM-score was 0.774 for SSAlign and 0.715 for Foldseek. This is a descriptive analysis of the selected evaluation set rather than evidence of uniform performance across all protein lengths: the shortest bin contains only one query, and the 100 queries were selected according to the number of Foldseek hits.

To provide a membrane-protein example, we examined human neuropeptide Y receptor type 2 (NPY2R; UniProt P49146), a 381-residue class A G protein-coupled receptor. The four highest-ranked SSAlign candidates by cosine similarity were NPY2R-annotated proteins from mouse, bamboo partridge, elephant shark, and golden eagle; their *post hoc* TM-align avg_TM-scores ranged from 0.806 to 0.918 (Table 6, available as supplementary data at *Bioinformatics* online). The distribution of TM-align-evaluable hits is shown in Fig. 7, available as supplementary data at *Bioinformatics* online. Targets were identified by UniProt accession.

### 3.3 SSAlign retrieves simple-fold proteins not returned by Foldseek

Proteins composed of repetitive structural units, referred to here as simple-fold proteins, can be challenging for structure search. Foldseek achieves fast retrieval by compressing 3D contact maps into 1D 3Di sequences and searching them with an MMseqs2-based alignment strategy. This design is efficient, but it can reduce sensitivity for repetitive folds: 3Di uses only 20 discrete states to represent contact environments and relies on nearest-neighbor contacts. As a result, simple-fold proteins can collapse into repetitive patterns that are difficult to distinguish, and Foldseek’s *k*-mer prefilter can discard true homologs before alignment.

To evaluate this limitation, we identified 13 pairs of simple-fold proteins with pronounced repetitive structural fragments in the SCOPe40 database. Although these proteins are present in the database, Foldseek did not retrieve matches for them under the tested settings (Table 1, available as supplementary data at *Bioinformatics* online). By contrast, SSAlign retrieved most of these annotated relationships, indicating higher sensitivity on this set of simple-fold architectures.

Antimicrobial peptides (AMPs) provide another relevant test case. These small bioactive peptides often adopt simple α-helical conformations with repetitive, low-complexity structures and limited sequence conservation (Bahar and Ren 2013). We analysed seven representative AMPs from the Swiss-Prot database (P84381–P84387) and found that Foldseek did not retrieve homologs or self-alignments for several cases under the tested settings (Table 2, available as supplementary data at *Bioinformatics* online). In contrast, SSAlign retrieved most annotated pairs. As shown in Fig. 5, available as supplementary data at *Bioinformatics* online, when the AMP P84382 (XT-2) is used as the query, the top-ranked SSAlign hits share a similar short α-helical conformation in PyMOL superposition. UniProt annotations indicate a shared biological context: these hits are amphibian host-defense AMPs secreted by skin glands and associated with the gastrin/cholecystokinin family and Magainin subfamily; P84382 is annotated as strongly active against *Staphylococcus aureus* but weaker against *Escherichia coli*. In these cases, SSAlign retrieved cross-species candidates in low-complexity proteins with repetitive motifs, where discrete 3Di k-mer prefiltering may miss true positives. In this small case set, SSAlign retrieved additional functionally related yet sequence-diverse structural units among the tested simple-fold proteins.

## 4 Discussion and conclusions

SSAlign combines a SA PLM, an embedding normalization module, and vector search for large-scale protein structure retrieval. In our benchmarks, this combination reduced runtime relative to Foldseek while maintaining competitive alignment quality.

A central component of SSAlign is ERM, which reshapes the embedding space before vector retrieval. SaProt embeddings show inter-dimensional correlation and unequal variance, so some dimensions can dominate cosine similarity. ERM applies a linear transformation to reduce these effects and produce a more isotropic representation, which was associated with higher first-stage retrieval overlap in our benchmarks.

On AFDB50, SSAlign completed the 1000-query benchmark approximately two orders of magnitude faster than Foldseek under comparable settings. This runtime reduction lowers the computational cost of proteome-scale structural search.

In addition to speed, SSAlign retrieved several homologous or self-alignment cases among the tested proteins with repetitive motifs or simple folds that were not returned by Foldseek under the same settings. TM-align remained more precise for the very top-ranked alignments, whereas SSAlign is intended for fast first-pass search. Future work will focus on the SS-score predictor and the global alignment step in SAligner. Extension to more complex targets, including protein–protein complexes, will require separate evaluation.

A practical limitation is the memory required by the exact FAISS embedding index. For *N* float32 vectors of dimension *d*, raw vector storage requires 4*Nd* bytes, excluding identifiers, mappings, model parameters, working buffers, and operating-system memory. For the 53 665 860-entry AFDB50 index at 512 dimensions, the vectors alone require approximately 102.4 GiB. The corresponding raw storage is approximately 51.2, 25.6, and 12.8 GiB at 256, 128, and 64 dimensions, respectively. Thus, memory grows linearly with database size and retained dimension. Lower-dimensional embeddings reduce this cost, although their effect on retrieval quality must be assessed for each benchmark. Query encoding and dynamic-programming rescoring also increase with protein length, and multi-domain proteins, complexes, membrane proteins as a class, mainly alpha proteins as a class, and proteins outside the evaluated distributions require broader validation.

## Supplementary Material

btag789_Supplementary_Data

## Data Availability

The source code for SSAlign is available under an open-source license at https://github.com/ISYSLAB-HUST/SSAlign. The archived software version and test data used in this study are available at Zenodo (doi: 10.5281/zenodo.23432919). All datasets, intermediate processing results, and prebuilt indices are publicly available at the SSAlign download page.
