# Supplementary material for: SSAlign: fast and sensitive protein structure search at scale
Source: Bioinformatics. 2026 Oct 26;42(10):btag789. doi: 10.1093/bioinformatics/btag789 (PMC13722286; doi:10.1093/bioinformatics/btag789)
Supplement: Supplementary file 1 [file btag789_supplementary_data.pdf]

Supplementary Materials for
SSAlign: Fast and Sensitive Protein Structure Search at Scale

Simple fold protein cases

We identified 13 simple-fold protein cases in the SCOPe40 dataset for which Foldseek did not retrieve the annotated family or superfamily relationship under the tested settings. Supplementary Table 1 summarizes the number of retrieved relationships at the family and superfamily levels. These examples suggest that SSAlign can recover additional candidates in some simple-fold cases. Supplementary Table 2 reports the seven antimicrobial peptide (AMP) cases discussed in the main text; "/" denotes no retrieved match in Foldseek, and "1" denotes self-match only. SSAlign retrieved additional candidates for most of these AMPs, with the average avg_TM-score of the top 10 results listed.

Supplementary Table 1: Simple-fold protein cases in SCOPe40

	d1dpjb_	d2e74d	d1q90g	d1xoua_	d1ehkc_	d1l2paa_	d2ciob_	d1q90m_	d1ehkb2	d1jb0x_	d1jb0m_	d1q90a3	d1rzhh2
Super Family	1	4	1	2	1	1	3	2	4	1	1	1	3
Family	1	4	1	1	1	1	1	2	4	1	1	1	3
TM-align	/	/	/	/	/	/	/	/	/	/	/	/	/
SSAlign-prefilter-500	1	4	1	2	1	1	1	2	2	1	1	1	3
SSAlign	1	4	1	2	1	1	1	2	2	1	1	1	3

Supplementary Table 2: Antimicrobial peptide cases in Swiss-Prot

ID	3Di sequences	Foldseek	SSAlign
P84381	DPVPVVVVVVCVVVCVVPVVPPDDPPD	/	0.66
P84382	DVVVVVVVVVVVCVVVVVCCVVDVD	1	0.80
P84383	DVCVVVVVVVVCCVVPVVVVVVPPD	1	0.58
P84384	DVVVVVVVVCVVPVVVCVVPVD	1	0.53
P84385	DCPVVVVVVVVVVVCVVVVVVD	/	0.72
P84386	DVVVVCVVVVVVVVVVVD	/	0.69
P84387	DVCPVVVVVVVVVVVVVD	1	0.50

Structure-aware protein language model

We used SaProt because it provides sequence-structure protein representations suitable for structure-aware retrieval. For embedding layer selection, we randomly sampled a subset of Swiss-Prot for validation and compared embeddings from the 33rd layer with the average of the 1st and 33rd layers (Supplementary Table 3). We calculated the overlap rate between the top-k results from the SSAlign-prefilter stage and those returned by Foldseek. The 33rd-layer embeddings produced slightly higher overlap in this validation, so we used SaProt_650M_AF2 with this layer in the reported experiments.

Supplementary Table 3: Overlap rates with Foldseek for different SaProt embedding layers

layer	Top-k	Overlap Rate (%)
33	1,000	85.36
	2,000	89.94
	4,000	92.41
(33+1)/2	1,000	83.87
	2,000	88.89
	4,000	91.59

SS-score predictor

For each dataset, the predictor was fitted as SS-score = $a \times$ Cosine_Similarity $+ b$. The fitted coefficients are listed in Supplementary Table 4.

Supplementary Table 4: Dataset-specific coefficients of the linear SS-score predictor

Dataset	Slope (a)	Intercept (b)
Swiss-Prot	0.69	0.39
SCOPe40	0.59	0.42

Command-line parameters

Supplementary Table 5: Key SSAlign command-line parameters used for database search

Parameter	Default / setting	Description
`db`	Swiss-Prot, SCOPe40, AFDB50	Target database to search.
`querypdbs`	user supplied	Comma-separated query structure files; each query is searched independently.
`prefilter_target`	2000	Number of candidates retained after FAISS-based SSAlign-prefilter retrieval.
`prefilter_threshold`	0.3	Score cutoff used to select marginal candidates for second-stage SAligner rescoring.
`max_target`	1000	Maximum number of final homologs reported per query.
`mode`	1	Execution mode; `0` runs SSAlign-prefilter only, and `1` runs prefilter plus SAligner rescoring.
`prefilter_mode`	cpu / gpu	Backend for FAISS retrieval; GPU mode offloads retrieval and can shard the index across GPUs.
`out_dir`	user supplied	Output directory for search results and intermediate files.
`nproc`	64	Number of CPU threads or processes used for parallel SAligner execution.
`cuda_device`	e.g. `cuda:0`	CUDA device used for SaProt inference.

NPY2R membrane-protein case study

Human neuropeptide Y receptor type 2 (NPY2R; UniProt P49146) was used as a query against AFDB50. Supplementary Table 6 shows the four highest-ranked candidates by native cosine similarity, and Supplementary Fig. 7 summarizes the distribution of hits with valid post hoc TM-align results. The avg_TM-score and RMSD values are evaluation metrics rather than native SSAlign output scores.

Targets were identified by UniProt accession. This example illustrates result interpretation for a membrane-protein search.

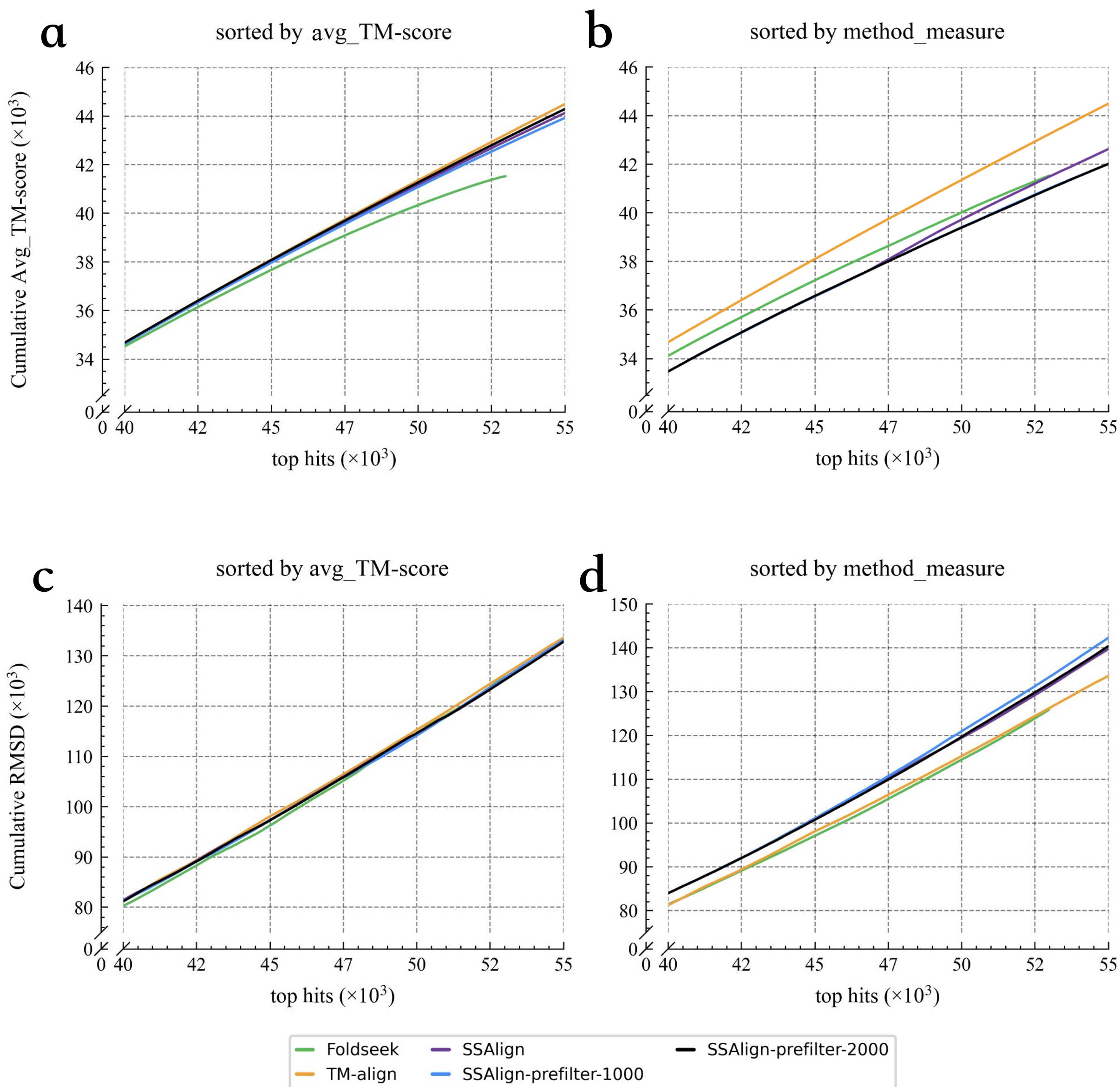

Supplementary Figure 1: **Comparative performance of structural alignment methods on the Swiss-Prot dataset.** The benchmark used 100 query proteins and a database of 542,378 entries. (a,c) Cumulative avg_TM-score and RMSD for five methods when results are ordered by TM-align-derived avg_TM-score. (b,d) The same evaluation when results are ordered by each tool's native score (E-value or SS-score). TM-align results were filtered with avg_TM-score ≥ 0.5. Foldseek curves end earlier because Foldseek returned fewer hits than the other methods.

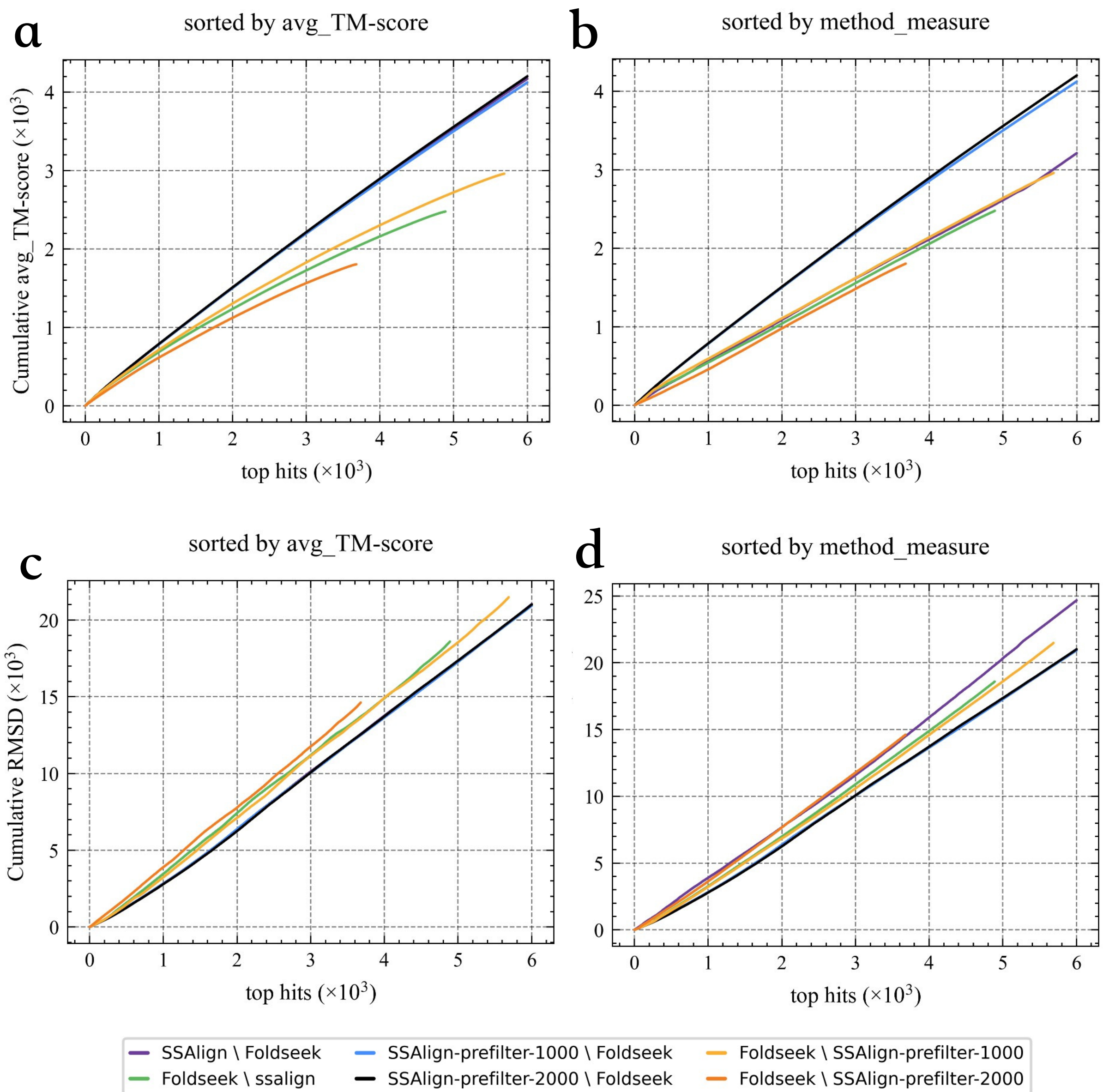

Supplementary Figure 2: **Differential matches between Foldseek and SSAlign on the Swiss-Prot dataset.** Using the same 100 query proteins, we compared the differential hit sets returned by Foldseek and SSAlign/SSAlign-prefilter. (a,c) Cumulative avg_TM-score and RMSD for six differential sets ordered by avg_TM-score. (b,d) The same sets ordered by each tool's native score (E-value or SS-score). The legend indicates protein pairs retrieved exclusively by one method.

Supplementary Table 6: Representative SSAlign retrieval results for the human NPY2R query

Rank	Target accession	Annotation	Organism	Cosine similarity	avg_TM-score	RMSD (Å)
1	P97295	NPY2R	mouse	0.6567	0.9180	2.26
2	A0A2P4T4T7	NPY2R	bamboo partridge	0.6252	0.8059	2.58
3	A0A4W3I7C9	NPY2R	elephant shark	0.6158	0.8296	2.25
4	A0A663F9S0	NPY2R	golden eagle	0.6148	0.8310	2.17

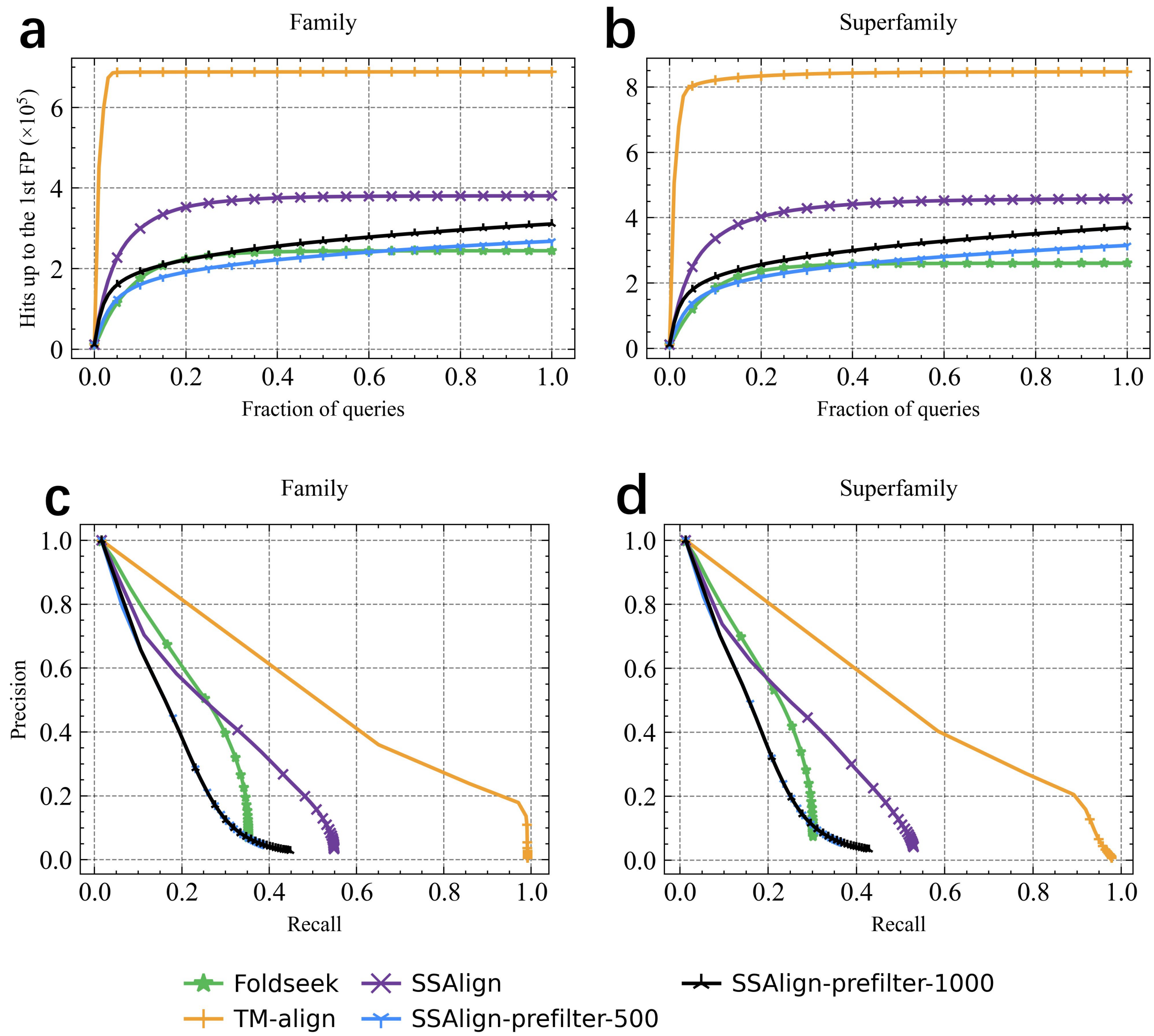

Supplementary Figure 3: **All-against-all evaluation on the SCOPe40 dataset.** True positives (TPs) were defined as proteins in the same family or superfamily, or pairs with avg_TM-score ≥ 0.5. False positives (FPs) were pairs from different folds with avg_TM-score < 0.5. (a,b) Fraction of TPs retrieved before the first FP for each method. (c,d) Precision–recall curves. TM-align results were obtained by all-against-all search and ranked by avg_TM-score. Because each query returned 11,211 results, TM-align reached full recall.

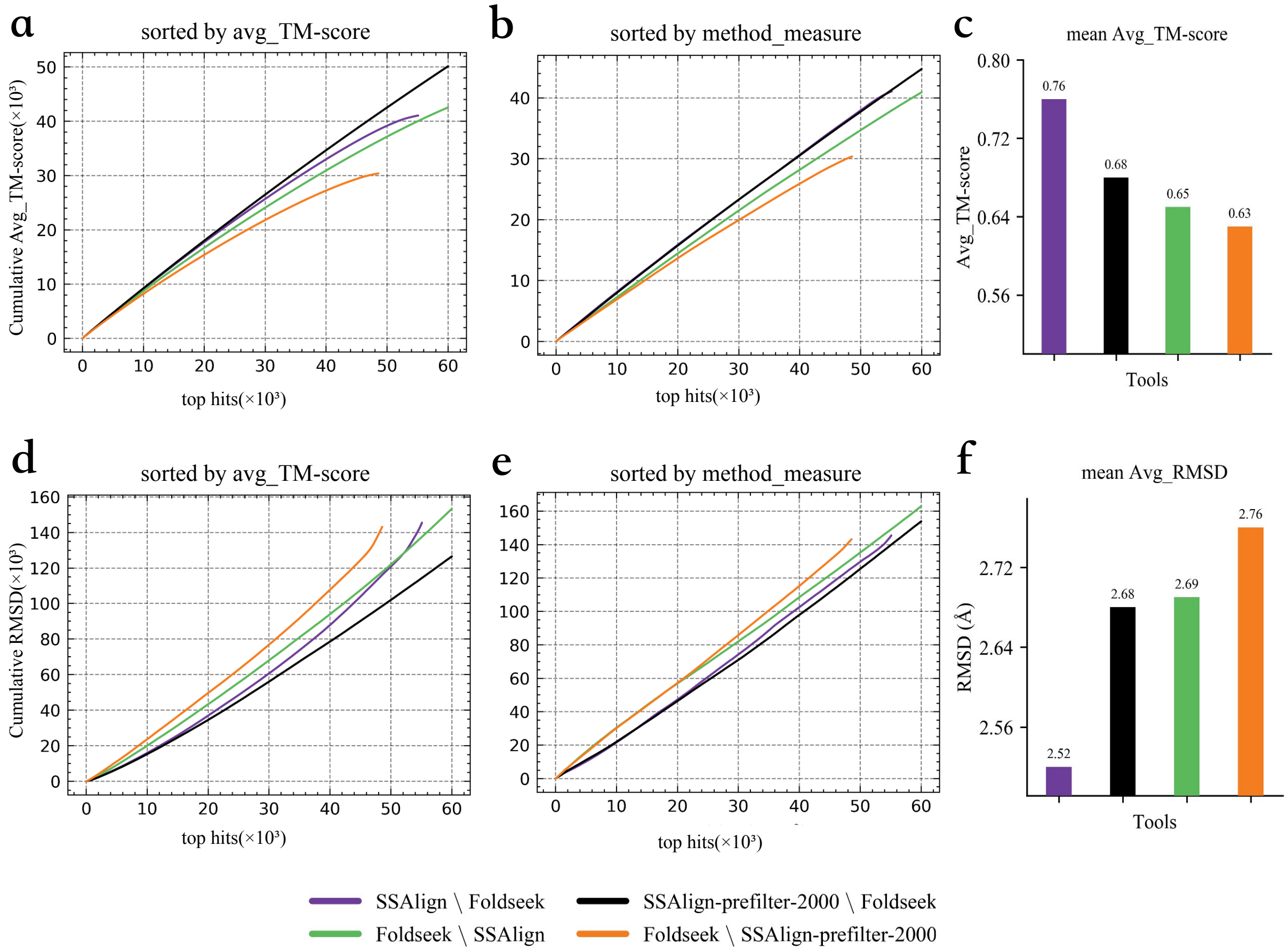

Supplementary Figure 4: **Differential matches between Foldseek and SSAlign on the AFDB50 dataset.** Using the same 100 query proteins, we compared differential hit sets returned by Foldseek and SSAlign/SSAlign-prefilter-2000. (a,d) Cumulative avg_TM-score and RMSD for six differential sets ordered by avg_TM-score. (b,e) The same sets ordered by each tool's native score (E-value or SS-score). (c,f) Average scores for the differential sets across the 100 queries. The legend indicates protein pairs retrieved exclusively by one method.

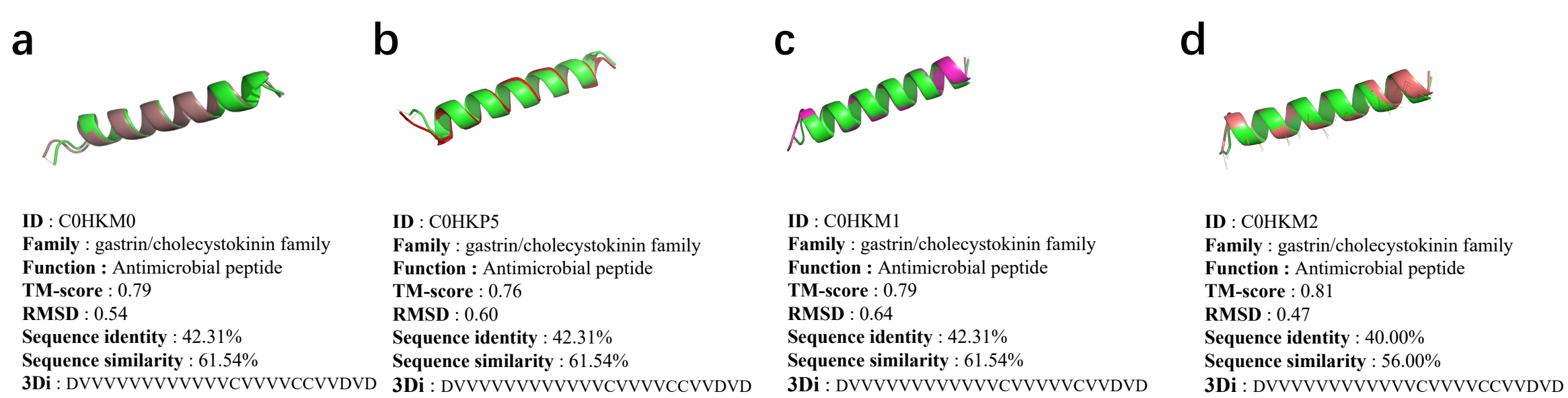

Supplementary Figure 5: **Structural superposition of the top-ranked SSAlign hits (C0HKM0, C0HKP5, C0HKM1, and C0HKM2) with the AMP query P84382 (XT-2).** All hits share a short α-helical fold and high structural similarity (RMSD ≈ 0.47–0.65 Å; avg. TM-score ≈ 0.764–0.814).

Additional benchmark and case-study figures

Prefilter threshold

Supplementary Fig. 6 illustrates the effect of different `--prefilter_threshold` values on Swiss-Prot retrieval performance under a fixed `--prefilter_target 2000`.

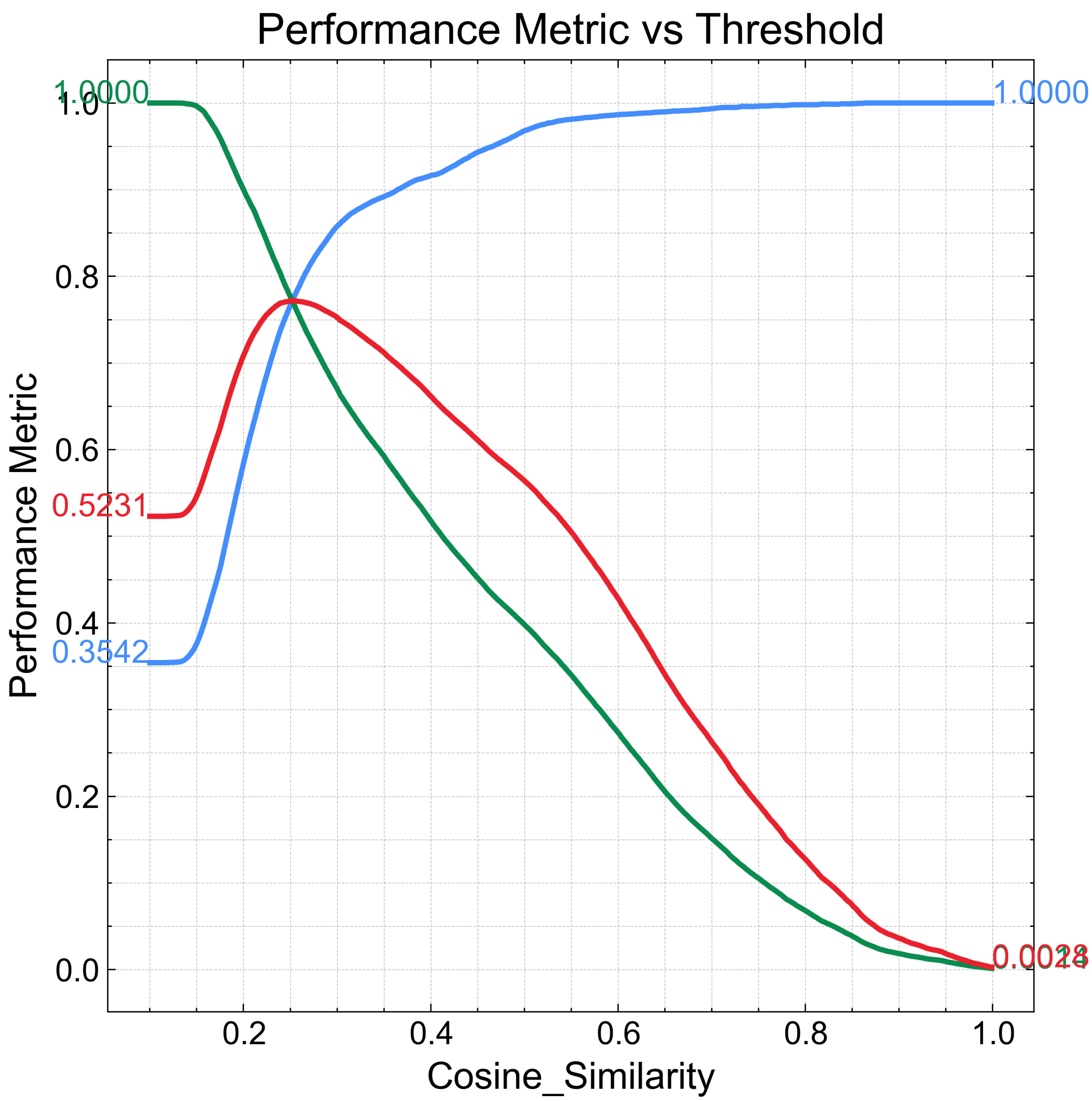

Supplementary Figure 6: **Effect of `-prefilter_threshold` on Swiss-Prot retrieval.** Precision (blue), recall (green), and F1 score (red) are plotted against the cosine-similarity threshold for 512-dimensional embeddings with `-prefilter_target 2000`. In the reported experiments, `-prefilter_threshold 0.3` was used as the default setting.

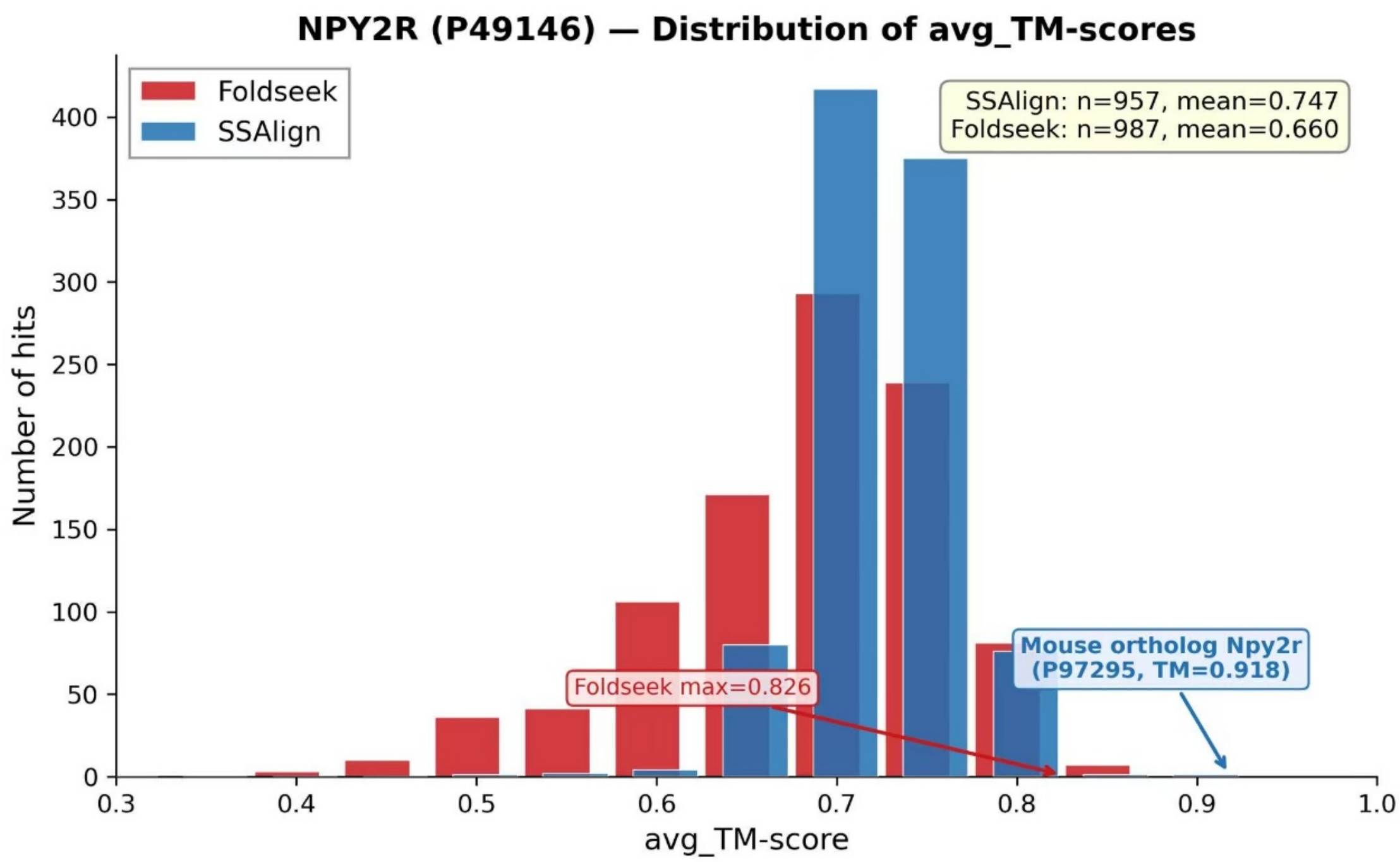

Supplementary Figure 7: **Distribution of TM-align-derived avg_TM-scores for the human NPY2R query against AFDB50.** Histograms include hits with valid TM-align results (SSAlign, $n = 957$; Foldseek, $n = 987$). The annotated maxima are 0.918 for SSAlign and 0.826 for Foldseek; the SSAlign maximum corresponds to mouse Npy2r (UniProt P97295), which Foldseek did not retrieve under the tested settings. Values were calculated post hoc with TM-align and are evaluation metrics rather than native search scores.
